# Mapping the current trends and hotspots of adult hippocampal neurogenesis from 2004–2023: a bibliometric analysis

**DOI:** 10.3389/fnins.2024.1416738

**Published:** 2024-06-18

**Authors:** Ye Liu, Jian Zhang, Xiyao Gu, Shushan Jia

**Affiliations:** ^1^The Second School of Clinical Medicine of Binzhou Medical University, Yantai, Shandong Province, China; ^2^Department of Anesthesiology, Department of Radiology, Renji Hospital, Shanghai Jiao Tong University School of Medicine, Shanghai, China; ^3^Key Laboratory of Anesthesiology (Shanghai Jiao Tong University), Ministry of Education, Shanghai, China; ^4^Department of Anesthesiology, The International Peace Maternity and Child Health Hospital, Shanghai Jiao Tong University School of Medicine, Shanghai, China

**Keywords:** adult hippocampal neurogenesis, neurogenesis, web of science, bibliometric analysis, Citespace, VOSviewer, co-citation analysis

## Abstract

**Objective:**

We utilized bibliometric and data visualization techniques to discern the primary research domains and emerging frontiers in the field of adult hippocampal neurogenesis (AHN).

**Methods:**

We systematically searched the Web of Science database for AHN-related articles published between 2004 and 2023. The retrieved articles were filtered based on publication types (articles and reviews) and language (English). We employed CiteSpace, VOSviewer, and the online bibliometric platform (bibliometric.com) to visualize and analyze the collected data.

**Results:**

In total, 1,590 AHN-related publications were discovered, exhibiting a steady increase in yearly publications over time. The United States emerged as the leading contributor in AHN research in terms of both publication quantity and national influence. Among all research institutions in the field of AHN, the University of California System exhibited the highest impact. Kempermann, Gerd was the most active author. The publications of the top three active authors primarily focused on the functions of AHN, and reversing hippocampal damage and cognitive impairment by improving AHN. An analysis of reference co-citation clustering revealed 8 distinct research clusters, and the notable ones included “adult hippocampal neurogenesis,” “neurogenesis,” “hippocampus,” “dentate gyrus,” “neural stem cell,” and “depression.” Additionally, a burst keyword detection indicated that ‘anxiety’ is a current research hotspot in the field of AHN.

**Conclusion:**

This in-depth bibliographic assessment of AHN offers a deeper insight into the present research hotspots in the field. The association between AHN and cognitive diseases, such as Alzheimer’s disease (AD) and anxiety, has emerged as a prominent research hotspot.

## 1 Introduction

The process of adding new neurons to the hippocampal region throughout life is referred to as adult hippocampal neurogenesis (AHN) (Kempermann et al., 2015). For decades, AHN has been extensively studied in rodents and non-human primates (Zhang et al., 2023), and many studies have also determined the extent of lifelong neurogenesis in humans (Spalding et al., 2013; Boldrini et al., 2018; Sorrells et al., 2018). Hippocampal neural stem cells in the subgranular zone (SGZ) of the dentate gyrus (DG) primarily differentiate into dentate granule neurons, which constitute the majority of excitatory neurons in the DG region (Denoth-Lippuner and Jessberger, 2021). Functionally, the DG receives input from the entorhinal cortex and sends information to CA3 and CA1 through a trisynaptic circuit, playing a key role in learning and memory (Babcock et al., 2021). AHN is the most robust form of plasticity in the adult brain and may contribute to the development of memory (Goncalves et al., 2016). Studies have shown that AHN enhances the plasticity of the hippocampus, which may be related to spatial memory, emotional memory, pattern separation, cognitive flexibility, and emotional regulation. Abnormal AHN can disrupt cognition, leading to memory deficits (Altman, 1963). Considering the importance of AHN for healthy brain function, many studies deciphered the physiological mechanisms of AHN.

Abnormal AHN has been well-documented in various diseases of the central nervous system, including neurodegenerative and neuropsychiatric disorders (Lazarov and Hollands, 2016). The morphology of adult-born dentate granule cells was abnormal in autopsy specimens of patients with various neurodegenerative diseases, such as amyotrophic lateral sclerosis, Huntington’s disease, Parkinson’s disease, Lewy body dementia, and frontotemporal dementia. In addition, changes were observed in the expression of differentiation markers of these cells. The proportions of hippocampal neural stem cells change in the resting and proliferative phases, altering the dynamic balance of the neurogenic niche. These changes reduce the phagocytic ability of microglia, trigger astrogliosis, and alter the microvascular structure of the DG, leading to hippocampal dysfunction in the physiological and pathological aging process of humans (Terreros-Roncal et al., 2021). Several studies have shown that AHN is reduced in patients suffering from depressive disorders such as Major Depressive Disorder (MDD) (Berger et al., 2020). MDD is a prevalent mental illness, primarily manifested as anxiety, low spirits, and slowed cognition. Studies on human post-mortem brain tissue have shown a decrease in the number of hippocampal neurons in patients with MDD and reduced angiogenesis in the neurogenic niche (Boldrini et al., 2012). These changes may be the main reasons for abnormal regulation of emotion, depression, and anxiety. Whether AHN has other potential functions, in addition to cognitive function and emotional regulation, still requires further scientific research.

Research on the mechanism of AHN primarily focuses on the initiation and maintenance of AHN. The neurogenic niche plays a crucial role in the production and functional maintenance of AHN. The composition of niche cells includes neural stem cells (NSCs), neural progenitor cells, neuroglial cells, endothelial cells, and immune cells, such as microglia and macrophages (Li et al., 2022). Under steady-state conditions, NSCs provide signaling molecules, such as the neurotransmitter GABA and the Notch ligand Delta-like 1 (Dll1) (Kawaguchi et al., 2013), through autocrine and paracrine manners to regulate the dormant state of NSCs. Microglia phagocytose apoptotic IPCs (Sierra et al., 2010), and astrocytes release cytokines, such as IL-1β and IL-6, to promote the neuronal differentiation of NSCs (Barkho et al., 2006). The presence of antibody plaques and tau tangles in the brain of patients with AD, and even the interaction of amyloid peptides with hippocampal niche macromolecules, such as palmitic acid and metals, can lead to chronic inflammation, oxidative stress, microglial activation, and abnormal function of astrocytes (Babcock et al., 2021). This affects NSC survival, neuronal differentiation, and the maturation and integration of newborn neurons (Li et al., 2022). Considering the importance of the neurogenic niche in the generation and maintenance of AHN changes in niches and their effect on AHN in different diseases need further in-depth research.

Bibliometric analysis utilizes statistical techniques to measure numerical data associated with scientific endeavors. It provides structured knowledge systems by handling the characteristics of references like journals, authors, institutions, and so on. Nevertheless, quantitative approaches are rarely employed in the field of AHN. We analyzed AHN studies to assess global research patterns and prospective areas of interest from 2004 to 2023. Additionally, we forecasted the direction of research in this domain for the upcoming years.

## 2 Methods

### 2.1 Data source and search strategy

On March 15, 2024, we completed the search on the Web of Science Core Collection database (Science Citation Index Expanded (SCI-EXPANDED) – 1900-present). The search strategy was: ‘Adult hippocampal neurogenesis’ (Title) or ‘Adult hippocampal neurogenesis’ (Abstract) or ‘Adult hippocampal neurogenesis’ (Author keywords). The search spanned from January 1, 2004, to December 31, 2023. Detailed information, such as publication types (articles or reviews), author details (full names, addresses, and affiliations), citation data (times cited in Web of Science core collection and all databases), and publication characteristics (year, date, volume, issue, page range, DOI, PubMed ID, research areas, and open access designations), were collected from the Web of Science database on the same day. All analyses were performed using the acquired data to prevent biases arising from citation information updates.

### 2.2 Data extraction and statistical analysis

The dataset for this study comprised the full records of the retrieved papers, such as titles, authors, abstracts, and cited references. These data were exported as plain text. We analyzed the number and year of publication, total citations, average citations per publication, countries/regions, institutions, authors, references, and keywords. Data were then uploaded to the Online Analysis Platform of Literature Metrology,^1^ VOS viewer (Version 1.6.20, Leiden University, Netherlands,), and CiteSpace (Version 6.2.R4, 64-bit, Drexel University, Philadelphia, PA, United States) for bibliometric analysis.

CiteSpace is specialized visual analysis software designed to analyze trends and dynamic changes in scientific literature. It identifies key points within a given field (Chen and Song, 2019). In this study, CiteSpace was used to analyze the collaborative relationships and co-citation of institutions, cited authors, subject categories, and references and detect citation bursts for references. We initiated our analysis by setting suitable parameters, which included the width of the time slice and the threshold. To simplify the network, we opted for the “Pathfinder Network Scaling Algorithm.” The “Logarithmization” option was employed to maintain a balanced link distribution within the network. Furthermore, we activated the “Burst Citation Detection” option, a method specifically designed to detect emerging trends in scientific literature.

VOS viewer is a software used to construct bibliometric networks of the scientific literature (Yeung et al., 2020). In this study, we employed this software to analyze and visualize co-occurrence networks of keywords. The relatedness in co-occurrence analysis was determined based on the number of documents, in which keywords occurred together. Different nodes represent different elements in the network maps generated by the VOS viewer, while the size of nodes is proportional to the number of publications or frequency. The color of nodes and lines indicates different appearance years or clusters. The line between the nodes represents the associations such as co-authorship or co-citation. The strength of links was assessed by the indicator of total link strength. When visualizing the results, we chose the “Cluster View,” which can group keywords based on their co-occurrence patterns.

The Online Analysis Platform of Literature Metrology (see text footnote 1) is a web-based tool used to examine the yearly publication trends of the top ten most productive countries/regions and explore collaborations among countries. We first uploaded our dataset, then selected “Country/Region” and “Year” as the dimensions for analysis. We used the “Collaboration Network Analysis” feature to explore the collaboration relationships between different countries/regions. We also looked at the results of the “Publication Trend Analysis” to understand the changes in the number of publications and citations in each country/region.

By employing these diverse bibliometric tools, we aimed to thoroughly analyze and illustrate data related to AHN. Our findings offer valuable insight into the trends and movements in the field.

## 3 Results

### 3.1 Bibliometric analysis of publications

**Figure 1:**
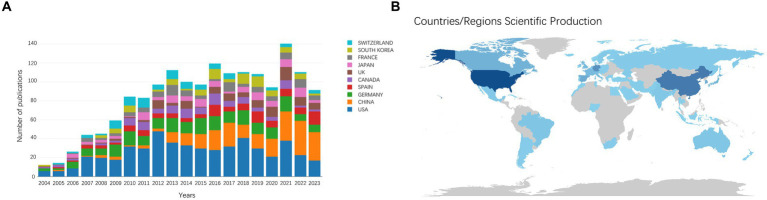
**(A)** The annual number of publications in the top 10 most productive countries from 2004 to 2023. **(B)** A world map depicting the contribution of each country/region based on publication counts.

The number of publications and citations within a specific period directly reflects the development trend of scientific knowledge in a particular field. We identified 1,590 original and review articles for bibliometric analysis. The annual changes in publications related to AHN research are shown in Figure 1A. Although the number of AHN publications occasionally fluctuates, it was generally on an upward trajectory, peaking in 2021 (132 publications). The difference between 2004 and 2021 (13 and 132, respectively) indicated that AHN is increasingly attracting global attention. In total, 64 countries/regions participated. Figure 1B depicts a world map illustrating the contribution of each country. The color gradient on the map corresponds to the number of publications. The publications mainly came from North America, East Asia, and Western Europe. More specifically, the United States contributed nearly one-third of the publications (522, 32.83%), almost three times that of mainland China (230, 14.47%) and Germany (218, 13.71%) (Table 1). Meanwhile, these publications have received 93,004 citations, with an average of 58.49 citations per publication. The increase in the number of publications and citations highlights the prospects and significance of AHN research.

**Table 1:** Top 10 countries/regions and institution in terms of publications for AHN.

Ranking	Country/region	Publications	Ranking	Institution	Publications
1	USA	522	1	Helmholtz Association	100
2	China	230	2	Technische Universitat Dresden	78
3	Germany	218	3	Institut National de la Sante et de la Recherche Medicale (Inserm)	61
4	Spain	112	4	German Center for Neurodegenerative Diseases (DZNE)	53
5	Canada	102	5	Consejo Superior de Investigaciones Cientificas (CSIC)	53
6	France	97	6	University of Texas System	53
7	Japan	95	7	Humboldt University of Berlin	48
8	South Korea	88	8	Charite Universitatsmedizin Berlin	47
9	Switzerland	84	9	Free University of Berlin	47
10	UK	77	10	Columbia University	44

### 3.2 Basic knowledge structures of AHN research

#### 3.2.1 Collaborating countries/regions and institutions in AHN research

**Figure 2:**
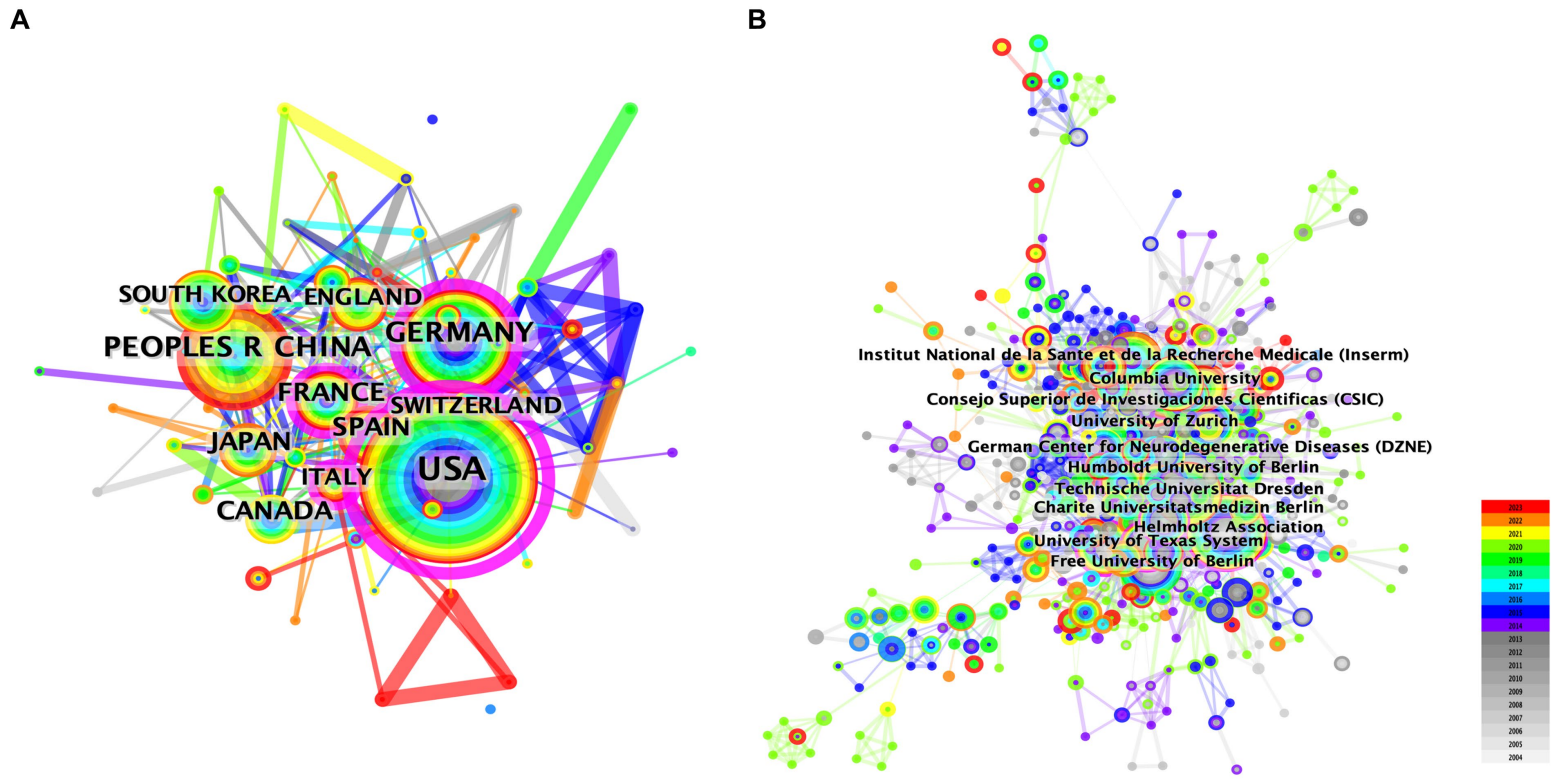
The network map of collaborating countries/regions **(A)** and institutions **(B)** in AHN research.

We conducted a country/region collaboration analysis using CiteSpace to reflect the collaborative relationships among countries/regions in the field of AHN. The analysis revealed 64 nodes and 314 edges, representing 64 countries/regions contributing to AHN research. The United States, France, Germany, and Italy were defined as centers for international cooperation and exchange with other countries (purple circles, Figure 2A). Notably, the distance between these circles was long, indicating a lack of cooperation between countries. “Centrality” is an important indicator used to describe network characteristics, reflecting the influence and importance of nodes within the network. Table 2 shows that the influence of the United States (0.70) was far greater than that of other countries/regions, followed by Germany (0.27) and Italy (0.18). Although the number of publications in China ranked second, China’s influence was only 0.06. China has shown rapid growth since 2012, and for the first time in 2021, China surpassed Germany in terms of the total number of papers, ranking second (Figure 1A). This trend suggests that the contribution of China may increase in the near future.

**Table 2:** Top 10 countries/region and institutions in terms of centrality for AHN.

Ranking	Country/region	Centrality	Ranking	Institution	Centrality
1	USA	0.70	1	University of California System	0.20
2	Germany	0.27	2	National Institutes of Health (NIH) – USA	0.16
3	Italy	0.18	3	University of Texas System	0.15
4	France	0.14	4	Harvard University	0.14
5	UK	0.10	5	University of Amsterdam	0.13
6	Spain	0.07	6	Helmholtz Association	0.12
7	Switzerland	0.07	7	Institut National de la Sante et de la Recherche Medicale (Inserm)	0.10
8	South Africa	0.07	8	Johns Hopkins University	0.10
9	Peoples R China	0.06	9	Chinese Academy of Sciences	0.09
10	Sweden	0.06	10	State University System of Florida	0.09

Additionally, the institutional graph contained 735 nodes and 3,284 edges, with a relatively low density (density = 0.0122). The results indicated that 735 institutions have contributed to this field, but research groups were relatively distributed among various institutions (Figure 2B). Tables 1, 2 comprehensively list the top 20 institutions with the most output and the greatest influence, along with their number of publications and centrality. The Helmholtz Association contributed the most publications, followed by Technische Universitat Dresden and Institut National de la Sante et de la Recherche Medicale (Inserm). The University of California System possessed the most influence (0.20), followed by the National Institutes of Health (NIH) – USA (0.16) and the University of Texas System (0.15). These three institutions were all from the United States, indicating a close cooperative relationship among various institutions in the United States. These findings also indicate that institutions in other countries/regions need to strengthen scientific cooperation.

#### 3.2.2 Analysis of authors

**Figure 3:**
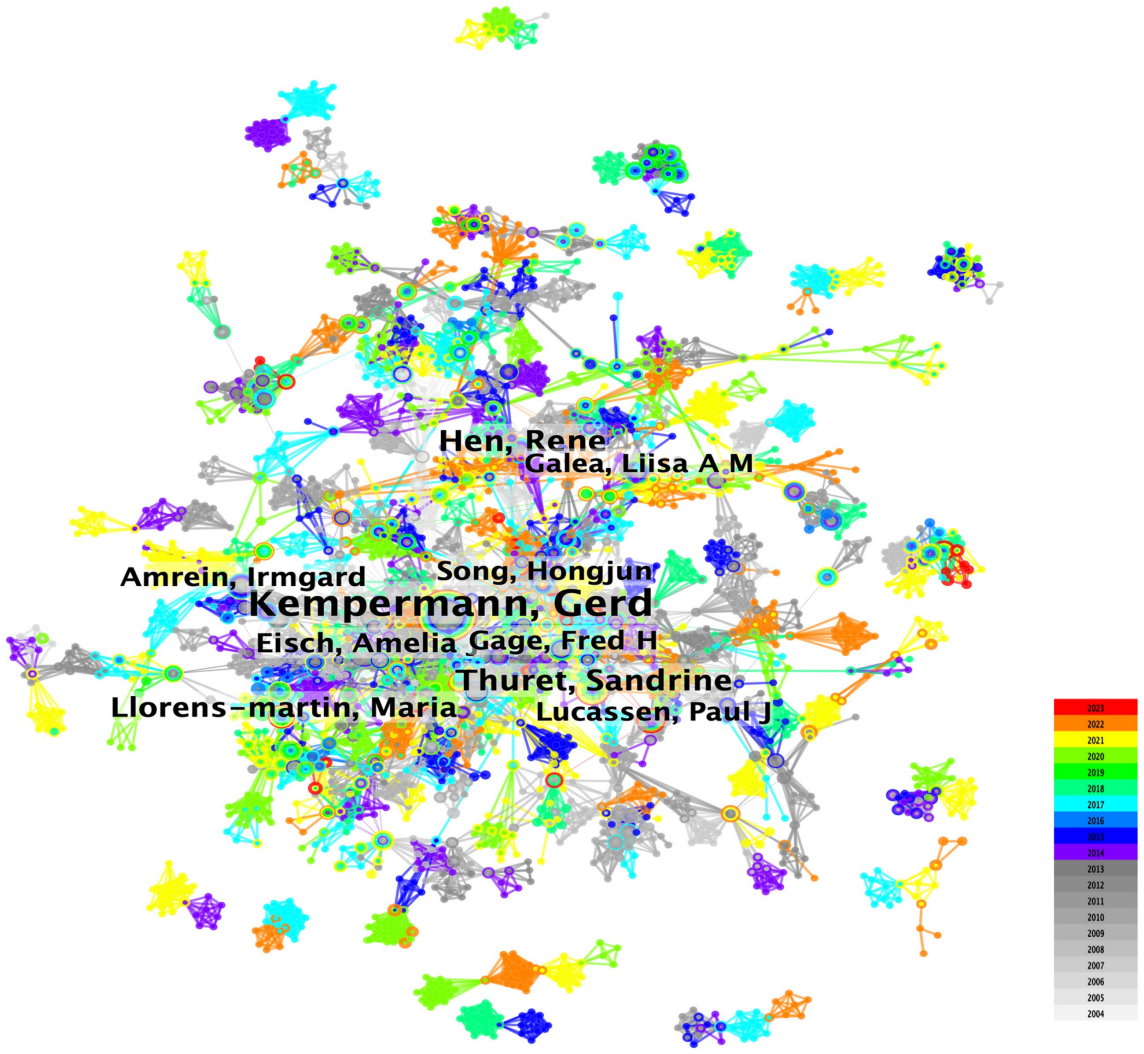
Overlay visualization map of author co-authorship analysis generated by VOSviewer software.

The author collaboration analysis identified 5,574 nodes and 19,982 edges, indicating that 5,574 authors contributed to 19,982 publications (Figure 3). The relatively low density (0.0013) suggests weak collaboration among authors in AHN research. Kempermann, Gerd (69), Hen, Rene (26), and Thuret, Sandrine (25) were the most active authors in terms of publication frequency (Table 3). Kempermann, Gerd initially focused on the functional significance of AHN (Kempermann et al., 2004; Kempermann, 2015) and existing evidence (Kempermann et al., 2018). His research is now focused on improving AHN, reversing hippocampal damage, and learning memory disorders (Leiter et al., 2022). Hen, Rene is dedicated to exploring the relationship between emotional disorders such as anxiety and depression (Santarelli et al., 2003; Samuels and Hen, 2011; Anacker and Hen, 2017; Jimenez et al., 2018), and neurogenesis. He has investigated the pathogenesis and therapeutic significance of adult major depression and loss of hippocampal neuroplasticity (Tartt et al., 2022). Thuret, Sandrine focused on diet (Vauzour et al., 2017), nutritional status (Marx et al., 2021), mental health (Zainuddin and Thuret, 2012), and hippocampal neurogenesis.

**Table 3:** Top 10 authors and cited authors in terms of publications for AHN.

Ranking	Author	Publications
1	Kempermann, Gerd	69
2	Hen, Rene	26
3	Thuret, Sandrine	25
4	Llorens-martin, Maria	21
5	Eisch, Amelia J	19
6	Amrein, Irmgard	18
7	Lucassen, Paul J	17
8	Song, Hongjun	17
9	Gage, Fred H	17
10	Galea, Liisa A M	16

### 3.3 Overview of research trends and hotspots

#### 3.3.1 Analysis of highly-cited studies

Citation analysis is the cornerstone of bibliometric analysis. Generally, a higher number of citations and citation frequency can reflect the strong influence and high academic value of a paper in a specific field. We listed the top 10 papers with the most citations and the highest centrality in the AHN field in Table 4. Notably, the papers written by Moreno-Jiménez EP had the most citations in this field, with 161 citations. Moreno-Jiménez EP and their colleagues obtained human brain samples under strict conditions. The authors found thousands of immature neurons in the DG of neurologically healthy human subjects, continuing to the ninth decade of life. These neurons showed varying degrees of maturity at different stages of AHN differentiation. The number and maturity of these neurons gradually declined with the progression of AD. These results indicate that AHN persists in physiological and pathological aging in humans and support the notion that impaired neurogenesis may be a potential mechanism for memory deficits in AD (Moreno-Jiménez et al., 2019). In 2013, Spalding KL and their colleagues published a paper with the highest citation in the centrality ranking in “Cell.” The authors assessed the generation of human hippocampal cells by measuring the concentration of nuclear bomb-test-derived ^14^C in genomic DNA and proposed an integrated model of cell turnover dynamics. The authors found that in adult humans, 700 new neurons were added to each hippocampus every day, equivalent to an annual turnover rate of 1.75% in the renewal site. The renewal process was slightly decelerated with aging. This result indicates that neurons continue to regenerate during adulthood, and the generation of adult hippocampal neurons may contribute to the function of the human brain (Spalding et al., 2013).

**Table 4:** Top 10 co-cited references related to AHN in terms of co-citations counts and centrality.

Ranking	Co-citation counts	Cited reference	Representative author (publication year)	Ranking	Centrality	Cited reference	Representative author (publication year)
1	161	Adult hippocampal neurogenesis is abundant in neurologically healthy subjects and drops sharply in patients with Alzheimer’s disease	Moreno-Jiménez et al. (2019)	1	0.2	Dynamics of hippocampal neurogenesis in adult humans	Spalding et al. (2013)
2	115	Human Hippocampal Neurogenesis Persists throughout Aging	Boldrini et al. (2018)	2	0.15	Young dentate granule cells mediate pattern separation, whereas old granule cells facilitate pattern completion	Nakashiba et al. (2012)
3	110	New neurons and new memories: how does adult hippocampal neurogenesis affect learning and memory?	Deng et al. (2010)	3	0.14	A functional role for adult hippocampal neurogenesis in spatial pattern separation	Clelland et al. (2009)
4	105	Human hippocampal neurogenesis drops sharply in children to undetectable levels in adults	Sorrells et al. (2018)	4	0.13	Requirement of hippocampal neurogenesis for the behavioral effects of antidepressants	Santarelli et al. (2003)
5	103	Adult hippocampal neurogenesis and cognitive flexibility—linking memory and mood	Anacker and Hen (2017)	5	0.12	Spatial relational memory requires hippocampal adult neurogenesis	Dupret et al. (2008)
6	99	Mechanisms and functional implications of adult neurogenesis	Zhao et al. (2008)	6	0.11	Increasing adult hippocampal neurogenesis is sufficient to improve pattern separation	Sahay et al. (2011)
7	96	Increasing adult hippocampal neurogenesis is sufficient to improve pattern separation	Sahay et al. (2011)	7	0.09	Enhanced synaptic plasticity in newly generated granule cells of the adult hippocampus	Schmidt-Hieber et al. (2004)
8	94	Dynamics of hippocampal neurogenesis in adult humans	Spalding et al. (2013)	8	0.09	Activation of local inhibitory circuits in the dentate gyrus by adult-born neurons	Drew et al. (2016)
9	93	Human Adult Neurogenesis: Evidence and Remaining Questions	Kempermann et al. (2018)	9	0.08	Human Hippocampal Neurogenesis Persists throughout Aging	Boldrini et al. (2018)
10	89	A functional role for adult hippocampal neurogenesis in spatial pattern separation	Clelland et al. (2009)	10	0.08	Neurogenesis-dependent and -independent effects of fluoxetine in an animal model of anxiety/depression	David et al. (2009)

#### 3.3.2 References co-citation analysis

**Figure 4:**
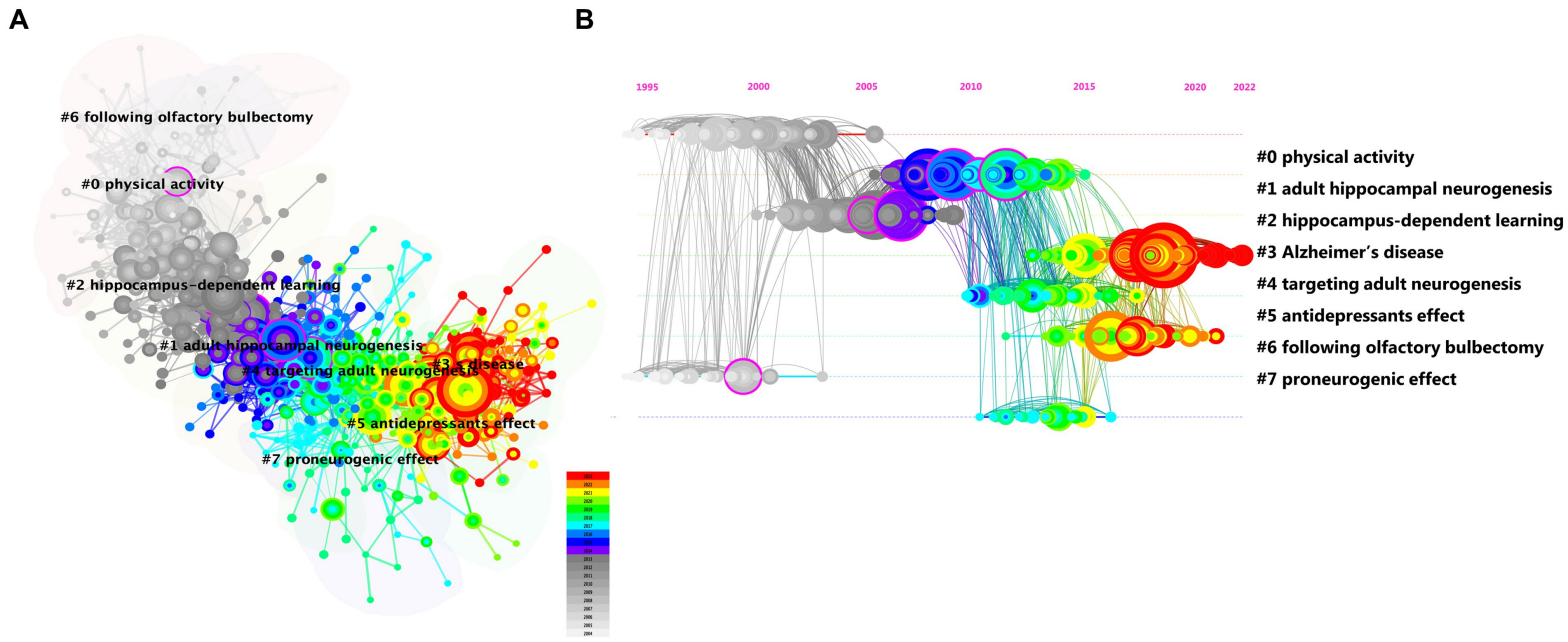
The cluster view map **(A)** and timeline view map **(B)** of reference co-citation analysis were generated by CiteSpace.

To identify milestone references and the focus of ongoing research, we conducted a co-citation analysis of references using CiteSpace and found eight major clusters (Figure 4A). We created a timeline view of these cluster evolutions to track the focus over time (Figure 4B). Early research focused on physical activity (#0), hippocampus-dependent learning (#2), and following olfactory bulbectomy (#6). As our understanding of adult neurogenesis deepened, the unique role of AHN or adult hippocampal neurogenesis in the hippocampal brain region was gradually discovered. People began to focus on investigating adult hippocampal neurogenesis (#1) and proneurogenic effect (#7). In addition, targeting adult neurogenesis (#4) also attracted widespread attention. Although the current focus on AHN is not particularly high, the approach of targeting AHN in various cognitive diseases will gradually become a hotspot and trend for future research. Currently, the most prominent trends include Alzheimer’s disease (#3) and antidepressant effects (#5). AHN abnormalities have been found in various cognitive diseases, and improvements in AHN can significantly improve the cognitive function of these diseases. Therefore, the current most prominent trend is that people are increasingly paying attention to the relationship between various diseases related to the hippocampal brain region and AHN. Among them, the most widely researched diseases are neurodegenerative diseases and neuropsychiatric diseases.

#### 3.3.3 Analysis of most frequently appearing keywords

**Figure 5:**
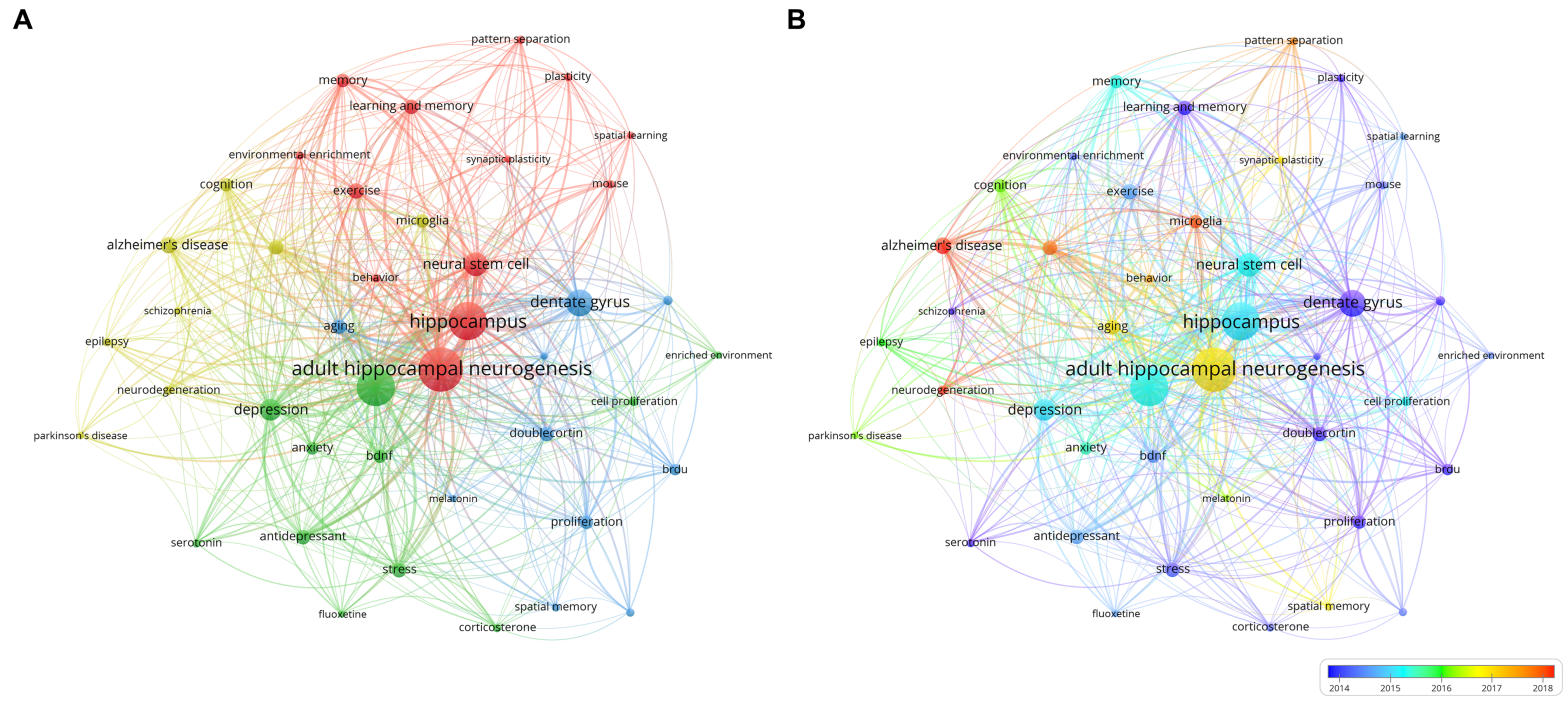
Overlay visualization map of keywords co-occurrence analysis in clusters **(A)** or in timeline **(B)**.

Keyword co-occurrence analysis is another common method in bibliometric analysis used To identify popular research topics. Co-occurrence analysis assesses the relevance between keywords based on the number of documents in which they appear together (Wu et al., 2021). In this study, we extracted author keywords from 1,590 publications and analyzed them using VOSviewer. Table 5 lists the 20 keywords with the highest frequency. In addition to “adult hippocampal neurogenesis,” The following keywords appeared more than 210 times: “Dentate gyrus,” “neurons,” “neural stem cells,” “brain,” and “synaptic plasticity.” As shown In The VOSviewer keyword co-occurrence visualization (Figure 5A), all keywords were divided into four different clusters and were marked. From major to minor, we summarized neurodegenerative diseases, neuropsychiatric diseases, neurogenic niches, and the potential functions of AHN. These common keywords were mainly linked to the role of AHN in various central nervous system diseases, cognitive functions, and the basic composition of neurogenic niches in AHN.

**Table 5:** Top 10 keywords in terms of frequency and centrality in AHN research.

Ranking	Keyword	Frequency	Ranking	Keyword	Centrality
1	Dentate gyrus	747	1	Cell-proliferation	0.11
2	Adult neurogenesis	419	2	Rat	0.08
3	Adult hippocampal neurogenesis	318	3	Enriched environment	0.08
4	Neurons	290	4	Messenger-rna	0.08
5	Neural stem cells	273	5	Mice	0.07
6	Brain	245	6	Cell proliferation	0.07
7	Hippocampal neurogenesis	240	7	Environmental enrichment	0.07
8	Synaptic plasticity	216	8	Depression	0.07
9	Proliferation	175	9	Antidepressant treatment	0.07
10	Progenitor cells	171	10	Element binding protein	0.07

Additionally VOSviewer color-coded the keywords based on their average year of appearance (Figure 5B). Keywords appearing early are represented in blue while those appearing recently are highlighted in red. Notably keywords such as "doublecortin" "proliferation" and "dentate gyrus" stood out in the early stages of research. In contrast keywords like "Alzheimer's disease" "microglia" and "neurodegeneration" showed a more recent average year of appearance. This is consistent with our findings from the co-citation analysis of references indicating that AHN is receiving increasing attention in the field of neurodegenerative diseases related to cognitive dysfunction and has become a hotspot in current research.

#### 3.3.4 Keywords and reference citation burst detection

**Figure 6:**
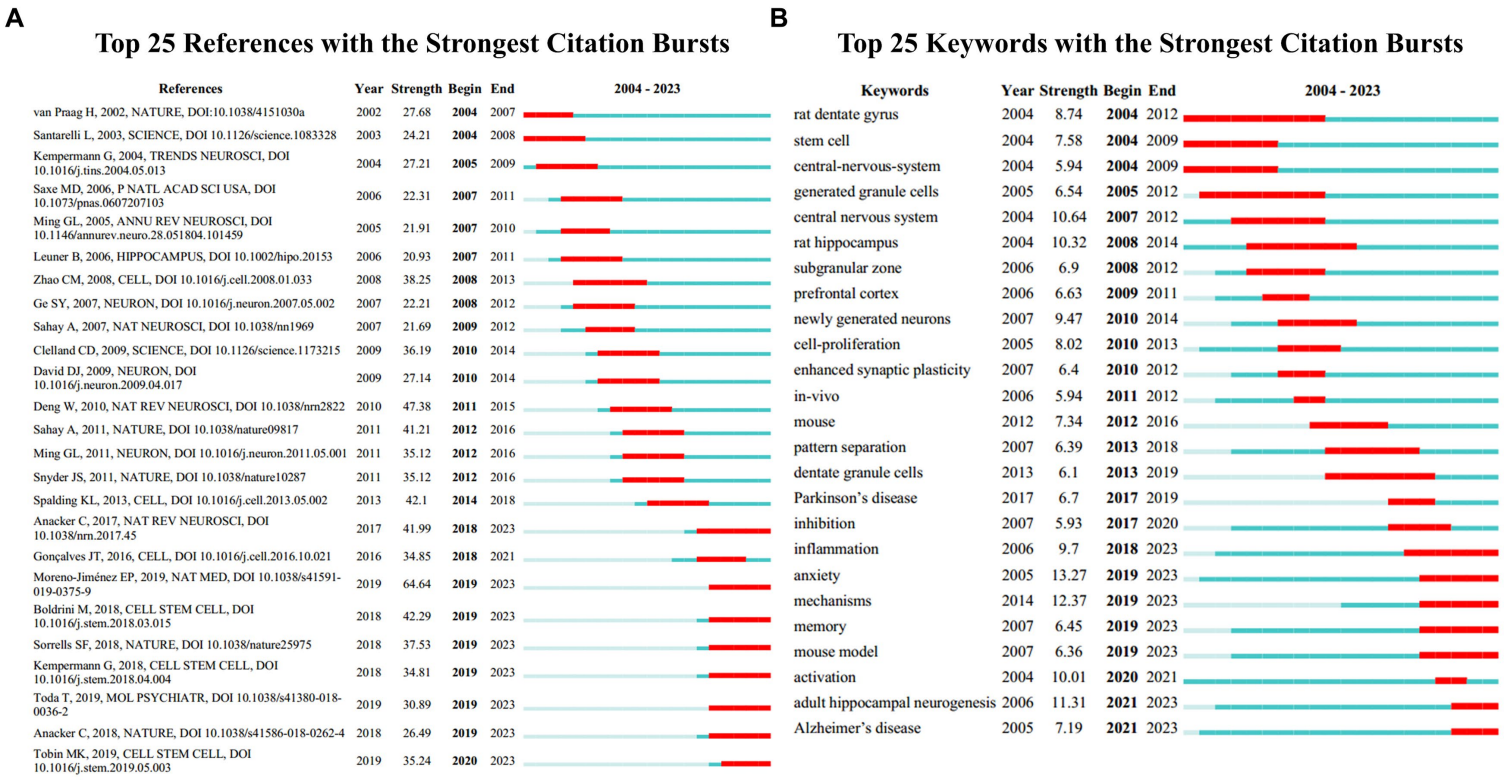
**(A)** References with the strongest citation bursts in publications on AHN research between 2004 and 2023. **(B)** Keywords with the strongest citation bursts in publications on AHN research between 2004 and 2023. The blue lines represent time intervals, while the red segments represent the periods when reference bursts occur.

Burst detection, an algorithm developed by Kleinberg, is an effective analytical tool for capturing concepts or topics widely discussed in a specific period. We applied burst detection to extract key references in the AHN research field. Figure 6A shows the top 25 references with the most significant citation bursts. In this graphical representation, the blue line represents the time interval, and the red segment represents the period when the reference burst occurred. Among these references, the one with the strongest burst value was an article written by Moreno-Jiménez et al. (64.64), which is also the most cited article in this field. This article had the strongest burst value and the most citations because the authors used human brain samples to provide direct evidence for the presence of AHN in the human brain, a question that has long been controversial. The authors also compared the brain samples of AD patients with neurologically healthy subjects, providing evidence for neural damage as a potential mechanism for cognitive dysfunction. Following closely, the reference with the second highest burst value was a review published by Deng et al. (2010) (47.38). The authors summarized the different contributions of newborn neurons at different maturity stages in learning and memory (Deng et al., 2010). In addition, although the burst of some references gradually weakened, 8 references showed continuous bursts. This finding indicates that these research topics have been receiving attention in recent years. We summarized these 8 references in a continuous burst state, mainly covering the role of AHN in aging, AD, health, and other disease states, and providing evidence for the existence of AHN (Anacker and Hen, 2017; Anacker et al., 2018; Boldrini et al., 2018; Kempermann et al., 2018; Sorrells et al., 2018; Moreno-Jiménez et al., 2019; Tobin et al., 2019; Toda et al., 2019).

Burst keyword detection is used to identify emerging concepts that are frequently cited over a period of time. In the past twenty years, “anxiety” ranked first with the highest burst intensity (13.27), followed by “mechanisms” (12.37), “adult hippocampal neurogenesis” (11.31), “central nervous system” (10.64), and “rat hippocampus” (10.32) (Figure 6B). Both “anxiety” and “mechanisms” began to burst in 2019 and their burst continued until 2023, accompanied by “memory” (6.45), “mouse model” (6.36), “activation” (10.01), and “Alzheimer’s disease” (7.19), indicating that these are current research hotspots.

## 4 Discussion

### 4.1 Primary findings

This study used bibliometric methods to analyze 1,590 articles on AHN from 2004 to 2023. The number of publications in the field of AHN has increased over the past 20 years. The United States led other countries in terms of both the number of publications and centrality. However, the number of publications in the field of AHN in mainland China has been steadily increasing since 2012, and in 2021, it surpassed Germany for the first time, ranking second in terms of total publications. Among research institutions, the Helmholtz Association contributed the most publications, while the University of California System had the greatest influence. In terms of authors, Kempermann, Gerd was the most active author in terms of publication number. The main focus of these studies was on the evidence of AHN in the human brain and the relationship between abnormal AHN and cognitive impairment in AD. Analysis of clusters of commonly cited publications showed that the main research topics over the past 20 years have focused on the relationship between abnormal AHN and cognitive diseases, such as AD and depression, and treatments targeting AHN. Keyword analysis also illuminated that research on AHN and neurodegenerative diseases and neuropsychiatric diseases is currently a hot topic in the field of AHN.

### 4.2 Results of the study in context

#### 4.2.1 The current academic situation of countries/regions and institutions regarding AHN research

Analysis of countries and institutions showed that the United States and Europe were undoubtedly the leaders in the field of AHN research, with the United States leading in terms of both the number of publications and centrality. The early initiation of AHN research in the United States and Europe has led to the current trend. This outcome could be attributed to the substantial research investment in the AHN field by the United States and Europe, as well as their prolific contributions to the field of health sciences. The number of publications in China has grown rapidly over the past twenty years, and in 2021 it jumped to second place. However, in terms of the centrality of AHN, the influence of China was weak. Although, the growth trend of publications in the field of AHN in China indicates the attention and exploration of Chinese scientific researchers in the field of AHN, and it is believed that in the near future, the central position of China in the field of AHN will significantly improve. In addition, international cooperation in AHN research is currently limited, and cooperation is mainly concentrated in the United States and some European countries. Based on the low density of cooperation analysis, we suggest that research institutions should eliminate academic barriers, strengthen exchanges, and promote the progress of AHN research.

#### 4.2.2 AHN exists in the human brain and is closely related to cognitive functions

AHN exists in both rodent and human brains and is closely related to cognitive functions. Researchers, such as Kempermann G, have made significant contributions to this field, providing evidence for the existence of AHN and its function in the brain. Using human brain samples, Moreno-Jiménez and Spalding not only confirmed the existence of AHN in the adult human brain but also measured changes in neurons with aging and evaluated the process of cell turnover integration. Their work has greatly advanced the progress of scientific research in the field of AHN, providing a theoretical basis for the existence of AHN and confirming the contribution of adult hippocampal neurogenesis to human brain function.

Neurons can inherently generate action potentials. Electrophysiological studies of adult dentate gyrus indicated that this ability appears rapidly (within a week) after mitosis. For a period of time thereafter, new granule cells exhibit an excitatory rather than inhibitory response to the typical inhibitory neurotransmitter GABA (Chancey et al., 2013), suggesting that these cells are more likely to be excited to generate action potentials, while mature granule cells are strongly inhibited by GABA (Amaral et al., 2007; Pelkey et al., 2017). Adult-generated granule neurons also display other unusual electrophysiological characteristics that disappear as they reach full maturity, including enhanced synaptic plasticity and a lower threshold for LTP induction (Ge et al., 2007). The high plasticity of adult-generated neurons allows playing a unique role in functions associated with the hippocampus. Different parts of the hippocampus have different functions, with the dorsal hippocampus supporting spatial navigation learning and memory, and the ventral hippocampus being crucial for anxiety and stress regulation (Fanselow and Dong, 2010). Reports indicated that a reduction number of new neurons impairs cognitive performance in hippocampal-dependent tests, including object recognition memory, contextual fear conditioning, and spatial learning and memory in the Morris water maze, Barnes maze, and radial arm maze (Cameron and Glover, 2015). Rodents lacking new neurons exhibit impairments in social behavior. Adult neurogenesis in the hippocampal region is necessary for stress-induced social avoidance (Lagace et al., 2010) and social recognition memory (Cope and Gould, 2019).

Although researchers have observed abnormalities in AHN in various diseases through animal models of cognitive disorders, these abnormalities can manifest in several ways: a decrease in the number of newborn neurons, abnormal morphology and growth patterns, and an inability to integrate into the existing hippocampal spatial network structure (Richetin et al., 2017; Torres-López et al., 2023). Researchers have found that correcting these AHN abnormalities can improve cognitive function, but the weight of these abnormal AHN phenotypes in cognitive dysfunction remains unknown. Therefore, some scholars believe that AHN is not a key factor in these cognitive disorders, but there is a certain correlation. We believe that the reason for this controversy is due to the current lack of understanding of AHN. Although research on adult hippocampal neurogenesis was mentioned as early as the 1960s, the detailed mechanism of AHN has not been fully elucidated to this day due to the lack of tools and the difficulty in defining cell phenotypes. However, it is gratifying that with the application of transgenic technology and multiphoton microscopy, researchers can not only label AHN-related cell populations with fluorescent proteins, but also use real-time imaging systems to continuously monitor the neural activity of neurons in the DG region (Corenblum et al., 2016; Danielson et al., n.d.). In addition, the progress of technologies such as single-cell RNA sequencing, spatial metabolomics, proteomics, and interactomics (Alexandrov, n.d.; Dueñas and Lee, n.d.) provides powerful visualization tools and new perspectives for the regulation of neurogenesis, promoting our understanding of complex adult neurogenesis. The advancement of these technologies greatly aids our understanding of the relationship between abnormal AHN and cognitive function, as well as the research of future intervention treatments.

#### 4.2.3 The major trends and advantages of studies related to AHN

Cluster analysis and burst detection analysis are valuable tools for determining the general research trends in the field of AHN. In keyword cluster analysis, we found that common keywords mainly focus on the role of AHN in various central nervous system diseases. In burst detection, we found that literature and keywords in a continuous burst state focus on the relationship between AHN and aging, AD, health, and other diseases. Therefore, we inferred that one of the current research focuses in the field of AHN is exploring the mechanistic role of AHN in the development of cognitive diseases, such as depression and AD. AD is the most common type of dementia, and patients with AD show functional impairments in memory and cognitive functions. Some studies reported that adult hippocampal neurogenesis and neuronal maturation are inhibited in patients with AD, while gliogenesis increases in these patients (Hollands et al., 2017). Several mouse models indicated that adult neurogenesis is impaired in the hippocampal region in AD, and newly proliferated granule neurons fail to integrate into the existing network, leading to spatial memory deficits (Richetin et al., 2017). In addition, in human brain specimens and rodent experiments in various neurodegenerative diseases, such as Parkinson’s disease, frontotemporal degeneration, vascular dementia, and aging, there are reports of reduced numbers and functional impairments of newly generated neurons. These abnormalities are closely related to declined cognitive ability (Hattiangady and Shetty, 2008; Nuber et al., 2008; Cho et al., 2015; Kim et al., 2017).

“Anxiety” is the keyword with the highest intensity in keyword burst analysis, making it a hotspot in the field of AHN research. AHN is widely described as a key function of the hippocampus, highly sensitive to the effects of stress. Environmental challenges, such as unpredictable chronic mild stress, prenatal stress, chronic social defeat, early life stress, and glucocorticoid application, all impair hippocampal neurogenesis (Kessler et al., 1985; McEwen, 2004; Czéh et al., 2007; Dagytė et al., 2009). Adult cells in the ventral DG appear to be particularly susceptible to these stressors (Schoenfeld and Cameron, 2015). Prolonged stressors may create a vicious cycle, where stress damages neurogenesis, low neurogenesis fails to alleviate stress, and further loses neurons born in adulthood. Similar effects have also been observed in non-human primates, where social isolation or subordinate status reduces neurogenesis and leads to anhedonia (Perera et al., n.d.). In dealing with stress, reducing or enhancing neurogenesis can regulate anxiety-like and depression-like behaviors (Anacker and Hen, 2017). Therefore, from a clinical perspective, neurogenesis has significant implications for the development of emotional and anxiety disorders, and increasing neurogenesis may be a potential therapeutic strategy to promote the reversal of emotional disorders and cognitive flexibility, for the treatment of patients with depression and anxiety, which may be one of the reasons why “anxiety” has become a hotspot in the field of AHN research.

Given the close relationship between AHN and cognitive impairment diseases, we believe that therapies targeting AHN will become the research prospect in this field. In basic research, researchers have demonstrated the beneficial effects of drug intervention, growth factor stimulation of neural stem cell proliferation, exercise, and other methods on improving AHN and cognitive disorders (Cui et al., 2015; Norevik et al., 2023). However, these therapeutic methods from basic research are still in the preclinical research stage, and the translation of experimental results mainly obtained from mice to the human brain still faces challenges due to species differences, genetic background, gender, and age. Therefore, safe and efficient treatment strategies targeting AHN will become a hot topic in future AHN research. In addition, how does AHN lead to cognitive decline? Are the abnormalities of AHN the same in different diseases? What is the detailed mechanism of abnormal AHN, and are there any intervention targets for abnormal neurogenesis? For the prospect of medical career and the development of human health, these questions are also worth more rigorous and systematic clinical and basic research work to explore deeply.

## 5 Limitations

This study does have some limitations. First, this study solely focused on publications from the Web of Science core literature database, which means that many pertinent publications from other databases, like Medline, Scopus, and the Cochrane Library, were not included. Second, our study only included English publications, excluding publications from other languages. This might result in an underrepresentation of the contributions from non-English publications. Third, the CiteSpace analysis, which is based on the number and structural changes of citations and the theory of information fitness, is influenced by the quantity and structural changes of citations. It was a ‘citation-based’ tools to uncover the potential trends and hotspots, thus may not fully represent the true quality of articles.

## 6 Conclusion

This comprehensive bibliometric analysis reports the current state of research on AHN, providing insights into current trends and hotspots. This study indicated that the relationship between AHN and cognitive diseases, such as AD, depression, etc., has become a prominent research hotspot. Further studies on the mechanisms of abnormal AHN and treatments targeting AHN can contribute to the prevention and treatment of cognitive diseases.

## Data availability statement

The original contributions presented in the study are included in the article/supplementary material, further inquiries can be directed to the corresponding authors.

## Author contributions

YL: Writing – original draft, Writing – review & editing, Conceptualization, Data curation, Formal analysis, Investigation, Methodology, Project administration, Software, Supervision. JZ: Data curation, Formal analysis, Methodology, Project administration, Supervision, Validation, Writing – original draft, Writing – review & editing, Software. XG: Conceptualization, Data curation, Formal analysis, Funding acquisition, Investigation, Methodology, Project administration, Resources, Software, Supervision, Validation, Visualization, Writing – review & editing, Writing – original draft. SJ: Data curation, Funding acquisition, Methodology, Project administration, Resources, Software, Supervision, Writing – review & editing, Writing – original draft.

## Footnotes

^1^https://bibliometric.com/
